# Associação entre Ácido Úrico Sérico e Pré-Hipertensão e Hipertensão entre Adultos Chineses

**DOI:** 10.36660/abc.20200098

**Published:** 2021-06-08

**Authors:** Lijun Zhu, Xiaoyu Zhang, Zhengmei Fang, Yuelong Jin, Weiwei Chang, Yan Chen, Yingshui Yao

**Affiliations:** 1 School of Public Health Wannan Medical College Institute of Chronic Disease Prevention and Control Wuhu China Department of Epidemiology and Biostatistics, School of Public Health , Wannan Medical College / Institute of Chronic Disease Prevention and Control , Wuhu - China; 2 Department of Clinical Nutrition Hefei BOE Hospital Hufei China Department of Clinical Nutrition , Hefei BOE Hospital , Hufei - China; 3 Department of Medicine Anhui College of Traditional Chinese Medicine Wuhu China Department of Medicine , Anhui College of Traditional Chinese Medicine , Wuhu - China

**Keywords:** Doenças Cardiovasculares/epidemiologia, Pressão Arterial, Hipertensão, Fatores de Risco, Ácido Úrico, Hiperuricemia

## Abstract

**Fundamento:**

O ácido úrico (AU), produto final do metabolismo dos nucleotídeos das purinas, participa dos processos de doenças metabólicas e cardiovasculares. Evidências experimentais sugerem que o ácido úrico é um mediador importante na resposta fisiológica ao aumento da pressão arterial.

**Objetivo:**

Avaliar a associação entre os níveis séricos de AU e pré-hipertensão e hipertensão em uma população chinesa.

**Métodos:**

Conduziu-se um estudo transversal entre março e setembro de 2017, e 1.138 participantes com idades entre 35 e 75 anos foram incluídos neste estudo, onde 223 normotensos, 316 pré-hipertensos e 599 hipertensos foram selecionados para avaliar a associação entre níveis séricos de AU e hipertensão. Considerou-se um valor de p<0,05 estatisticamente significativo.

**Resultados:**

Os níveis séricos de AU foram significativamente maiores no grupo pré-hipertensão e hipertensão em comparação com o grupo controle em toda a população (p<0,05 para todos). A análise quantitativa das características indicou níveis séricos de AU (2,92±0,81, 3,06±0,85, 3,22±0,98 mg/d) linearmente aumentados em mulheres normotensas, pré-hipertensas e hipertensas, com um valor de p de 0,008. Os níveis séricos de AU nos quartis correlacionaram-se positivamente com a PAD (p<0,05), principalmente em mulheres. Após o ajuste para idade, sexo, índice de massa corporal (IMC), glicose (GLI), colesterol total (CT), triglicerídeos (TG), colesterol HDL (lipoproteína de alta densidade), as razões de chances ( *odds ratios* — ORs) e intervalos de confiança (IC) de 95% da pré-hipertensão, dos níveis séricos de AU mais baixos (referentes) aos mais altos foram 1,718 (1,028–2,872), 1,018 (0,627–1,654) e 1,738 (1,003–3,010). Além disso, o segundo quartil dos níveis séricos de AU esteve significativamente associado à hipertensão, com uma OR (IC 95%) de 2,036 (1,256–3,298).

**Conclusões:**

O presente estudo sugere que níveis séricos mais elevados de AU estão positivamente associados à pré-hipertensão e hipertensão entre adultos chineses.

## Introdução

A prevalência de doenças cardiovasculares (DCV) vem aumentando rapidamente nas comunidades mundiais. A taxa geral de prevalência padronizada por idade de doenças cardiovasculares aumentou significativamente de 1990 a 2016 — 14,7% — e o número anual de óbitos por DCV aumentou de 2,51 milhões para 3,97 milhões na China. ^1^ A hipertensão arterial representa uma grande carga para a saúde pública mundial devido à sua alta prevalência, sendo um importante fator de risco para uma série de DCVs, incluindo acidente vascular cerebral, infarto do miocárdio, insuficiência cardíaca e insuficiência renal. ^2^ De acordo com o documento “Summary of report on cardiovascular diseases in China (2018)”, o número de pacientes hipertensos na China é de cerca de 245 milhões, e a taxa de prevalência de homens é maior do que de mulheres. ^3^ A hipertensão, um distúrbio altamente heterogêneo, é influenciada pela interação entre diversos fatores, como ingestão de sódio, álcool, tabagismo, excesso de peso e fatores genéticos. ^4^ Nos últimos anos, muitos estudos mostraram que níveis elevados de ácido úrico (AU) sérico estão associados ao aumento da incidência de hipertensão. ^5 , 6^

O AU é o produto final do metabolismo do nucleotídeo da purina, e o distúrbio do metabolismo da purina ou a excreção anormal do AU pode levar ao aumento dos níveis séricos de AU. Além disso, o aumento da concentração sérica de AU no corpo resulta em hiperuricemia, levando à ocorrência de gota. ^7^ Um estudo de coorte mostrou que a hiperuricemia é preditor de hipertensão em homens e mulheres. ^8^ Pesquisas com animais revelaram que a hiperuricemia leve causa hipertensão e lesão renal em ratos por meio da estimulação do sistema renina-angiotensina e inibição da óxido nítrico-sintase neuronal (nNOS). ^9^ Como fator de relaxamento derivado do endotélio, o óxido nítrico é crucial para a manutenção da pressão arterial (PA). ^10^ Uma revisão sistemática e uma meta-análise verificaram que para um aumento de 60 umol/L nos níveis séricos de AU, o risco relativo de hipertensão aumentou em 13%, e esse risco parece mais pronunciado em indivíduos mais jovens e em mulheres. ^11^

A hiperuricemia está comumente associada à pré-hipertensão em adultos. ^12^ O AU sérico também se mostrou um fator de risco independente para um padrão circadiano de hipertensão *non-dipper.* ^13^ Quanto mais alto o nível de AU sérico, mais difícil é controlar a pressão arterial ambulatorial noturna, a pressão arterial diastólica noturna e o pico de pressão arterial matinal. ^14^ Em um estudo inicial, não se observou hiperuricemia em 25–40% dos hipertensos não tratados e 75% dos indivíduos com hipertensão maligna. ^15^ No entanto, não se encontrou nenhuma associação independente entre os níveis séricos de AU ou risco de hipertensão incidente entre homens mais velhos. ^16^

Quando a hipertensão se complica com hiperuricemia, ambas causam e afetam uma à outra, o que agrava o desenvolvimento da doença. Portanto, apesar da associação entre AU sérico e hipertensão, seu mecanismo permanece obscuro. Assim, em nosso estudo, exploramos a associação entre altos níveis séricos de AU e hipertensão entre adultos chineses na província de Anhui, no norte da China.

## Métodos

### Desenho do estudo

Este estudo foi realizado de março a setembro de 2017 no Centro de Exame Físico de um Hospital Popular na Província de Anhui, no norte da China. Um total de 1.191 participantes com idades entre 35 e 75 foram incluídos neste estudo, incluindo 643 casos de hipertensão e 548 indivíduos normotensos. Indivíduos sem valor sérico de AU (n=53) foram excluídos. Por fim, 1.138 adultos, incluindo 223 normotensos, 316 pré-hipertensos e 599 hipertensos, foram selecionados para avaliar a associação entre os níveis séricos de AU e hipertensão. O protocolo de estudo foi aprovado pelo Comitê de Ética da Wannan Medical College.

### Coleta dos dados e medições

Cada participante passou por entrevista presencial e preencheu um questionário padrão incluindo características demográficas, histórico clínico e características de estilo de vida. Todas as informações foram coletadas por uma equipe de pesquisa treinada. No exame físico, todos os indivíduos tiveram sua altura, peso e pressão arterial (PA) medidos. O índice de massa corporal (IMC) foi calculado como o peso corporal (kg) dividido pela altura ao quadrado (m ^2^ ). Uma equipe de pesquisa bem treinada mediu a PA uma vez usando esfigmomanômetro eletrônico com o participante na posição sentada após pelo menos 5 minutos de repouso. Todos os indivíduos jejuaram durante a noite por pelo menos 10 horas antes da coleta de sangue. Amostras de sangue venoso de 5 ml foram coletadas para medir os níveis de colesterol total (CT), triglicerídeos (TG), colesterol HDL, colesterol LDL, glicose (GLI) e níveis séricos de AU. Os fumantes foram definidos como consumidores de cigarros que fumavam ao menos 20 cigarros por semana ou ao menos 3 meses por ano. Beber álcool no mínimo 2 vezes por semana ou no mínimo 6 meses por ano foi considerado consumo de álcool.

### Definição

A hipertensão foi definida como PAS≥140 mmHg e/ou PAD≥90 mmHg, ou uso de anti-hipertensivos, e a pré-hipertensão foi considerada PAS de 120–139 mmHg e/ou PAD 80–89 mmHg. ^17^ A hiperuricemia foi definida como níveis séricos de AU >4,75 mg/dL em homens e >4,04 mg/dL em mulheres. ^18^ Os níveis séricos de AU foram categorizados por quartis como ≤2,65, 2,66–3,24, 3,25–3,98 e ≥3,99 mg/dL.

### Análise de dados

A normalidade dos dados foi determinada pelo teste de Kolmogorov-Smirnov. Os dados quantitativos são resumidos como média e desvio padrão (média±DP) com distribuição normal; os dados qualitativos, como proporções. As diferenças de sexo nas características gerais foram analisadas usando o teste t pareado de Student para variáveis contínuas e o teste qui-quadrado (χ2) para variáveis categóricas. As diferenças para as variáveis entre os grupos foram determinadas por análise de variância (ANOVA) de um fator ou teste χ2, e as correções de Bonferroni foram usadas para comparações múltiplas. Além disso, aplicou-se a análise de regressão logística múltipla não condicional para estimar a relação entre AU e hipertensão. Realizou-se o teste do coeficiente de correlação de Pearson para avaliar as inter-relações entre as variáveis basais e os níveis séricos de AU. Utilizou-se o equipamento Epidata 3.1 (The Epidata Association, Odense, Dinamarca) para estabelecer bases de dados. Todas as análises estatísticas foram realizadas com o software SPSS 18.0 (SPSS Inc., Chicago, IL). Um p<0,05 bicaudal foi definido como estatisticamente significativo.

## Resultados

### Características dos participantes

Este estudo incluiu 1.138 indivíduos (223 controles, 316 pré-hipertensos e 599 hipertensos) com idades entre 35 e 75 anos. A Tabela 1 apresenta os dados demográficos e as características clínicas dos participantes. As características de colesterol LDL, creatinina, tabagismo e consumo de álcool não foram significativamente diferentes entre os grupos, enquanto idade, índice de massa corporal (IMC), glicose (GLI), colesterol total (CT), triglicerídeos (TG), colesterol HDL e AU apresentaram diferenças significativas. Os níveis séricos de AU (mg/dL) foram significativamente maiores no grupo pré-hipertensão (3,5±1,1) e hipertensão (3,4±1,1) em comparação com o grupo controle (3,2±1,0) em toda a população (p<0,05 para todos). Além disso, a prevalência de hiperuricemia foi de 10,3%, 17,1% e 17,0% em normotensos, pré-hipertensos e hipertensos, respectivamente.

**Tabela 1:** – Características demográficas de normotensão, pré-hipertensão e hipertensão

Característica	Normotensão (n=223)	Pré-hipertensão (n=316)	Hipertensão (n=599)	p
Idade (anos)	56,1±11,3	58,2±11,2	61,2±9,8 ^#*^	0,000
IMC (kg/m ^2^ )	22,3±2,8	23,3±2,9 ^#^	24,1±2,9 ^#*^	0,000
PAS (mmHg)	108,3±7,8	126,2±7,4 ^#^	148,2±19,2 ^#*^	0,000
PAD (mmHg)	69,7±5,9	79,5±6,5 ^#^	89,1±13,0 ^#*^	0,000
GLI (mmol / L)	5,6±1,6	5,6±1,6	6,0±2,2 ^#*^	0,002
CT (mmol/L)	4,4±0,9	4,5±0,9	4,7±1,1 ^#^	0,003
TG (mmol/L)	1,2±0,8	1,3±0,8	1,7±1,2 ^#*^	0,000
Colesterol HDL (mmol/L)	1,4±0,4	1,3±0,4	1,2±0,4 ^#*^	0,000
Colesterol LDL (mmol/L)	2,6±0,8	2,7±0,8	2,6±0,8	0,235
Creatinina (umol/L)	82,5±61,8	80,4±13,5	86,1±39,2	0,110
AU (mg/dL)	3,2±1,0	3,5±1,1 ^#^	3,4±1,1 ^#^	0,013
Tabagismo atual (n=362)	65 (29,1%)	95 (30,1)	202 (33,7%)	0,336
Etilismo atual (n=415)	68 (30,5%)	118 (37,3%)	229 (38,2%)	0,114
Prevalência de hiperuricemia (n=179)	23(10,3%)	54(17,1%)	102(17,0%)	0,046

PAS: Pressão arterial sistólica; PAD: Pressão arterial diastólica; GLI: Glicose; CT: Colesterol total; TG: Triglicerídeos; HDL: Lipoproteína de alta densidade; LDL: Lipoproteína de baixa densidade; AU: Ácido úrico. p: Todos os participantes dos grupos de normotensão, pré-hipertensão e hipertensão tiveram as variáveis analisadas por ANOVA de um fator ou teste do qui-quadrado. #: p<0,05 vs. Normotensão. *: p<0,05 vs. Pré-hipertensão.

**Figura 1:**
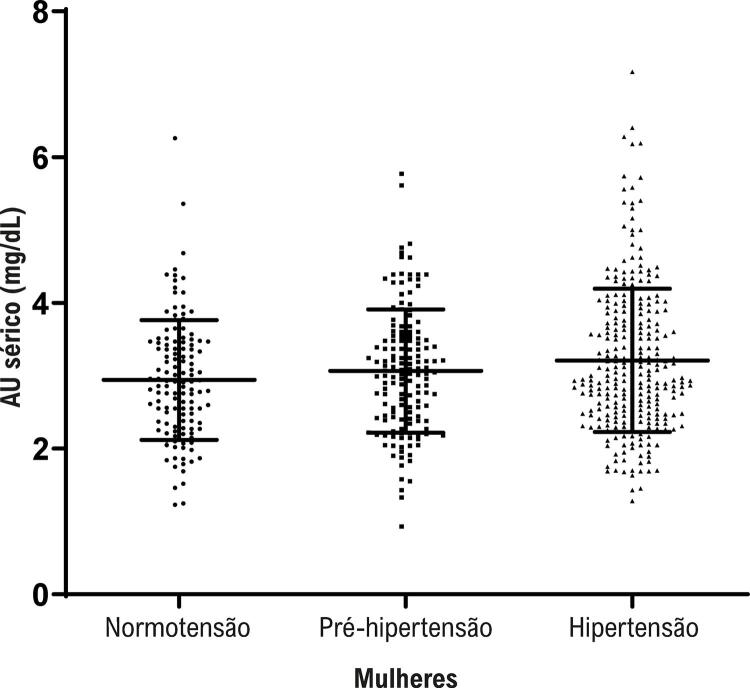
– Comparação dos níveis séricos de AU em normotensão, pré-hipertensão e hipertensão no sexo feminino.

Por subgrupo dividido por sexo, dos 1,138 indivíduos, 568 eram do sexo masculino e 570 eram do sexo feminino. O nível médio de AU sérico foi de 3,67 mg/dL em homens e 3,11 mg/dL em mulheres (p<0,05). Os níveis séricos de AU não mostraram diferença entre os grupos no sexo masculino. Outras análises quantitativas de características do AU sérico (mg/dL) indicaram que os níveis séricos de AU (2,92±0,81, 3,06±0,85, 3,22±0,98) aumentaram linearmente em normotensão, pré-hipertensão e hipertensão em mulheres, com valor de *p* de 0,008 ( Figura 1 ).

### Níveis de variáveis demográficas e clínicas nos quartis de AU sérico

A Tabela 2 apresenta as informações basais dos indivíduos em cada quartil de AU sérico. Os níveis médios de IMC, PAD, TG, colesterol LDL e creatinina mostraram-se aumentados com níveis elevados de AU sérico nos quartis (p<0,01 para tendência).

**Tabela 2:** – Características basais dos participantes do estudo de acordo com os quartis de AU sérico

Característica	AU sérico (mg/dL)				valor de p para tendência

	Q1(≤ 2,65)	Q2(2.66-3.24)	Q3(3,25-3,98)	Q4(≥3,99)	
Idade (anos)	60,2±10,5	59,2±10,5	58,7±10,7	59,4±11,1	0,305
IMC (kg/m ^2^ )	23,1±2,8	23,1±2,8	23,5±2,9	24,3±3,2	0,000
PAS (mmHg)	133,2±23,0	136,9±21,4	132,7±22,4	134,4±20,0	0,914
PAD(mmHg)	80,5±13,2	83,4±12,3	83,0±12,7	83,7±12,8	0,005
GLI (mmol / L)	6,0±2,4	5,8±2,2	5,6±1,5	5,8±1,5	0,120
CT (mmol/L)	4,5±1,0	4,5±1,0	4,5±0,9	4,7±1,00	0,098
TG (mmol/L)	1,2±0,7	1,3±0,9	1,5±1,1	1,8±1,3	0,000
Colesterol HDL (mmol/L)	1,3±0,4	1,3±0,5	1,2±0,4	1,2±0,4	0,266
Colesterol LDL (mmol/L)	2,5±0,8	2,6±0,8	2,7±0,9	2,9±0,8	0,000
Creatinina (umol/L)	73,7±14,3	81,9±53,5	83,1±14,1	96,6±54,4	0,000

*PAS: Pressão arterial sistólica; PAD: Pressão arterial diastólica; GLI: Glicose; CT: Colesterol total; GT: Triglicerídeos; HDL: Lipoproteína de alta densidade; LDL: Lipoproteína de baixa densidade; AU: Ácido úrico. p: Todos os participantes dos grupos de normotensão, pré-hipertensão e hipertensão tiveram as variáveis analisadas por ANOVA de um fator para tendência linear.*

### Correlação dos níveis séricos de AU e características clínicas por sexo

Os níveis séricos de AU estiveram positivamente correlacionados com IMC, pressão arterial diastólica (PAD), CT, TG, colesterol LDL e creatinina em ambos os sexos. Os níveis séricos de AU estiveram negativamente correlacionados com a idade e positivamente correlacionados com IMC, CT, TG, colesterol LDL e creatinina em homens. No sexo feminino, os níveis séricos de AU estiveram positivamente associados com IMC, PAD, TG, colesterol LDL e creatinina ( Tabela 3 ).

**Tabela 3:** – Correlação dos níveis séricos de AU e características clínicas dos participantes do estudo por sexo

Características	Homens		Mulheres		Total	

	r	p	r	p	r	p
Idade (anos)	-0,091	0,030	-0,042	0,314	-0,012	0,679
IMC (kg/m ^2^ )	0,167	<0,001	0,138	0,001	0,177	<0,001
PAS (mmHg)	-0,063	0,133	0.0.63	0,133	0,007	0,821
PAD (mmHg)	0,055	0,187	0,116	0,006	0,099	0,001
GLI (mmol / L)	-0,069	0,101	0,022	0,593	-0,017	0,570
CT (mmol/L)	0,152	<0,001	0,065	0,123	0,080	0,007
TG (mmol/L)	0,230	<0,001	0,205	<0,001	0,22	<0,001
Colesterol HDL (mmol/L)	0,011	0,786	0,001	0,998	-0,041	0,163
Colesterol LDL (mmol/L)	0,250	<0,001	0,148	<0,001	0,187	<0,001
Creatinina	0,143	0,001	0,443	<0,001	0,230	<0,001

*PAS: Pressão arterial sistólica; PAD: Pressão arterial diastólica; GLI: Glicose; CT: Colesterol total; TG: Triglicerídeos; HDL: Lipoproteína de alta densidade; LDL: Lipoproteína de baixa densidade; AU: Ácido úrico.*

### Associação entre quartis de AU sérico e pré-hipertensão e hipertensão

Na análise de regressão logística, a Tabela 4 apresenta as razões de chances de pré-hipertensão e hipertensão pelo aumento dos quartis de AU sérico. Após o ajuste para idade e sexo na pré-hipertensão, as razões de chances (ORs) (IC 95%) foram 1,686 (1,024–2,775) e 2,064 (1,220–3,492), respectivamente, no 2º e 4º quartil em comparação com o 1º quartil. Após ajustar novamente o IMC, GLI, CT, TG, colesterol HDL, a associação ainda se apresentava estatisticamente significativa. O segundo quartil dos níveis séricos de AU esteve significativamente associado à hipertensão, com um OR (IC 95%) de 2,061 (1,313–3,235) e 2,036 (1,256–3,298), para os modelos 1 e 2, respectivamente.

**Tabela 4:** – Associação entre quartis de AU sérico e pré-hipertensão e hipertensão

AU sérico (mg/dL)	Pré-hipertensão		Hipertensão	

	Idade ajustada para sexo	Multivariada	Idade ajustada para sexo	Multivariada
Q1(≤ 2,65)	1	1	1	1
Q2(2,66–3,24)	1,686 (1,024–2,775)*	1,718 (1,028–2,872)*	2,061 (1,313–3,235)*	2,036 (1,256–3,298)*
Q3(3,25–3,98)	1,091 (0,683–1,742)	1,018 (0,627–1,654)	1,105 (0,723–1,689)	0,912 (0,576–1,444)
Q4(≥3,99)	2,064 (1,220–3,492)*	1,738 (1,003–3,010)*	2,236 (1,387–3,606)	1,613 (0,967–2,690)

Multivariada ajustada para idade, sexo, IMC, GLI, CT, TG, Colesterol HDL; * Comparado com Q1, p<0,05.

## Discussão

Alterações nos níveis de AU estão envolvidas na remodelação vascular e na disfunção endotelial, que pode ser a causa de distúrbios cardiovasculares. ^19 , 20^ O AU pode ser considerado um importante antioxidante, que não só estabiliza a atividade da óxido nítrico sintase endotelial (eNOS), mas também aumenta o armazenamento de gordura e triglicerídeos. ^21^ Estudos epidemiológicos têm demonstrado forte associação entre o AU e a doença arterial coronariana, aterosclerose e hipertensão. ^22^ Em nosso estudo, relatamos que níveis séricos de AU mais elevados estiveram positivamente associados com pré-hipertensão e hipertensão na população de meia-idade e idosos, e níveis elevados de AU sérico podem indicar um aumento correspondente na PAD. O risco geral de pré-hipertensão aumentou 73,8% para o quartil mais elevado vs. mais baixo dos níveis séricos de AU, mesmo após o ajuste para possíveis variáveis de confusão. Além disso, verificamos que uma associação mais robusta nas mulheres participantes.

Estudos anteriores examinaram a associação entre os níveis séricos de AU e hipertensão, e os resultados estavam de acordo com nossos achados. Sundstrom et al., ^23^ revelaram que o aumento dos níveis séricos de AU foi preditor independente de desenvolvimento de hipertensão após um seguimento de curto prazo. Níveis elevados de colesterol LDL e AU sérico são fatores de risco para disfunção endotelial e envelhecimento vascular. A presença concomitante de valores subótimos de colesterol LDL e AU sérico está associada a risco aumentado de hipertensão em uma amostra populacional saudável. ^24^ Um estudo de coorte retrospectivo de 5 anos descobriu que o aumento de AU é um forte marcador de risco para hipertensão desenvolvida a partir da pré-hipertensão em adultos japoneses. ^25^ Além disso, estudos clínicos piloto sugerem que a redução dos níveis séricos de AU pode reduzir a pressão arterial em adolescentes pré-hipertensos. ^26^ Atualmente, a pré-hipertensão é comum na China. Aproximadamente 20–50% dos adultos são acometidos por pré-hipertensão em todo o mundo, e isso aumenta o risco de hipertensão incidente. ^27^ A prevalência de pré-hipertensão está aumentando rapidamente na China, mas suas causas e fatores associados não foram bem estudados.

Observamos que os níveis séricos de AU aumentaram linearmente na normotensão, pré-hipertensão e hipertensão em mulheres, e essa associação entre AU sérico e pressão arterial se mostrou mais forte entre as mulheres do que entre os homens. Além disso, os níveis séricos de AU estiveram associados positivamente à PAD, principalmente em mulheres. Alguns estudos anteriores demonstraram uma associação entre níveis séricos de AU e a hipertensão mais pronunciada em mulheres. Peng et al., ^28^ também verificaram que a hiperuricemia estava associada à pré-hipertensão entre 1.773 mulheres chinesas com idade ≥30. Resultados semelhantes foram apresentados em um estudo de seguimento, no qual Strasak et al., ^29^ demonstraram que o AU sérico é um preditor independente de todas as principais formas de morte cardiovascular em mulheres idosas. A variação dos níveis de AU sérico em mulheres na menopausa sugere uma interação com os hormônios sexuais. ^30^ Pesquisas mostram que a diferença de sexo na pressão arterial começa a aparecer na adolescência e o surto de crescimento puberal ocorre mais cedo nas meninas do que nos meninos. ^31^ Há aumento mais significativo da PAS em meninos do que em meninas, enquanto há maior aumento da PAD em meninas do que em meninos. ^32^ Outras alterações fisiológicas e hormonais complexas podem contribuir para a hipertensão.

Diversas limitações devem ser consideradas. Em primeiro lugar, o desenho transversal usado para avaliar a relação entre AU sérico e pré-hipertensão e hipertensão limita nossa capacidade de estabelecer uma relação causal. Este problema pode ser resolvido por estudos longitudinais no futuro. Em segundo lugar, o mecanismo de interação entre hipertensão e aumento de ácido úrico não foi explorado. Mais estudos ainda são necessários para examinar a possível diferença de sexo na associação entre os níveis séricos de AU e hipertensão em diferentes populações.

## Conclusões

Nossos achados sugerem que o AU sérico está significativamente associado à pré-hipertensão e hipertensão, e a associação se mostrou mais robusta nas participantes do sexo feminino. Portanto, o manejo precoce adequado dos níveis de AU em adultos pode ser importante para prevenir o desenvolvimento de hipertensão.
