# Supplementary material for: Associação entre Ácido Úrico Sérico e Pré-Hipertensão e Hipertensão entre Adultos Chineses
Source: Arq Bras Cardiol. 2021 Jun 8;116(6):1072–8. [Article in Portuguese] doi: 10.36660/abc.20200098 (PMC8288526; doi:10.36660/abc.20200098)
Supplement: Translation

# Translation: Association between Serum Uric Acid and Pre-hypertension and Hypertension among Chinese Adults

**Authors:** Lijun Zhu, Xiaoyu Zhang, Zhengmei Fang, Yuelong Jin, Weiwei Chang, Yan Chen, Yingshui Yao

**Affiliations:** 1 China Department of Epidemiology and Biostatistics, School of Public Health, Wannan Medical College/ Institute of Chronic Disease Prevention and Control, Wuhu - China; 2 China Department of Clinical Nutrition, Hefei BOE Hospital, Hufei - China; 3 China Department of Medicine, Anhui College of Traditional Chinese Medicine, Wuhu - China

**Keywords:** Cardiovascular Diseases/epidemiology, Blood Arterial, Hypertension, Risk Factors, Uric Acid, Hyperuricemia

## Abstract

**Background:**

Uric acid (UA), the end product of purine nucleotide metabolism, participates in the processes of metabolic and cardiovascular diseases. Experimental evidence suggests it is an important mediator in the physiological response to blood pressure increase.

**Objective:**

To evaluate the association between serum UA levels and pre-hypertension and hypertension in a Chinese population.

**Methods:**

A cross-sectional study was conducted from March to September 2017, and 1,138 participants aged 35 to 75 were enrolled in this study, where 223 normotensive, 316 pre-hypertensive, and 599 hypertensive subjects were selected to evaluate the association between serum UA levels and hypertension. A p-value <0.05 was considered statistically significant.

**Results:**

Serum UA levels were significantly higher in the pre-hypertension and hypertension group compared to the control group in the entire population (p<0.05 for all). Quantitative trait analysis indicated that serum UA levels were (2.92±0.81, 3.06±0.85, 3.22±0.98 mg/d) linearly increased in normotensive, pre-hypertensive and hypertensive females, with a p value of 0.008. Serum UA levels in the quartiles were positively correlated with DBP (p<0.05), particularly in females. After adjusting for age, gender, body mass index (BMI), glucose (GLU), total cholesterol (TC), triglycerides (TG), high-density lipoprotein cholesterol (HDL-c), the odds ratios (ORs) and 95% confidence intervals (CIs) of pre-hypertension from the lowest (referent) to the highest levels of serum UA were 1.718 (1.028–2.872), 1.018 (0.627–1.654) and 1.738 (1.003–3.010). Additionally, the second quartile of serum UA levels were significantly associated with hypertension, with an OR (95% CI) of 2.036 (1.256–3.298).

**Conclusions:**

This study suggests that higher serum UA levels are positively associated with pre-hypertension and hypertension among Chinese adults.

## Introduction

The prevalence of cardiovascular diseases (CVD) is increasing rapidly in the world communities. The overall age-standardized prevalence rate of cardiovascular diseases increased significantly from 1990 to 2016 — by 14.7% — and the annual number of deaths from CVD increased from 2.51 million to 3.97 million in China.^1^ High blood pressure (BP) has a major public health burden worldwide due to its high prevalence and it is a major risk factor for a series of CVD including stroke, myocardial infarction, heart failure and renal failure.^2^ According to the “Summary of report on cardiovascular diseases in China (2018)”, the number of hypertensive patients in China is about 245 million and the prevalence rate of males is higher than that of females.^3^ Hypertension, a highly heterogeneous disorder, is influenced by the interaction between many factors such as sodium intake, alcohol, smoking, overweight, and genetic factors.^4^ In recent years, many studies have shown that high serum uric acid (UA) levels are associated with increased incidence of hypertension.^5 , 6^

UA is the end product of purine nucleotide metabolism, and the disorder of purine metabolism or abnormal excretion of UA can lead to increased serum UA levels. Furthermore, increased serum UA concentration in the body results in hyperuricemia, ultimately leading to gout.^7^ A screened cohort study has shown that hyperuricemia is a predictor of hypertension in both men and women.^8^ Animal research has revealed that mild hyperuricemia causes hypertension and renal injury in rats via stimulation of the renin-angiotensin system and inhibition of neuronal nitric oxide (NO) synthase.^9^ As an endothelial-derived relaxing factor, NO is crucial to the maintenance of blood pressure (BP).^10^ A systematic review and meta-analysis reported that for a 60 umol/L increase in serum UA levels, the relative risk of hypertension increased by 13%, and this risk appears more pronounced in younger individuals and women.^11^

Hyperuricemia is commonly associated with pre-hypertension in adults.^12^ Serum UA has also been shown to be an independent risk factor for a non-dipper circadian pattern of hypertension.^13^ The higher the level of serum UA, the more difficult it is to control nighttime ambulatory blood pressure, nighttime diastolic blood pressure and morning blood pressure peak.^14^ In an early study, hyperuricemia was reported in 25–40% of untreated hypertensive and 75% of malignant hypertensive subjects.^15^ However, no independent association between serum UA levels and risk of incident hypertension was found among older men.^16^

When hypertension is complicated with hyperuricemia, both of them cause and affect each other, which aggravates the development of the disease. Therefore, despite an association between serum UA and hypertension, its mechanism remains unclear. Thus, in our study, we explored the association between high serum UA levels and hypertension among Chinese adults in Northern Anhui Province.

## Methods

### Study design

This study was conducted from March to September 2017 at the Physical Examination Center of a People’s Hospital in Northern Anhui Province. A total of 1,191 participants aged 35 to 75 were enrolled in this study, including 643 hypertension cases and 548 normotensive subjects. Individuals with missing serum UA (n=53) value were excluded. Ultimately, 1,138 adults, including 223 normotensive, 316 pre-hypertensive, and 599 hypertensive subjects, were selected to evaluate the association between serum UA levels and hypertension. The study protocol was approved by the Ethics Committee of Wannan Medical College.

### Data collection and measurement

Each participant completed a face-to-face interview and a standard questionnaire including demographic characteristics, medical history, and lifestyle characteristics. All information was collected by trained research staff. On physical examinations, all subjects were measured for height, weight, and blood pressure (BP). Body mass index (BMI) was calculated as body weight (kg) divided by height squared (m^2^). A well-trained research staff measured BP once using electronic sphygmomanometer with the participant in the sitting position after at least 5 minutes of rest. All the subjects fasted overnight for at least 10 hours before blood sampling. Venous blood samples of 5 ml were taken for measuring plasma total cholesterol (TC), triglyceride (TG), high-density lipoprotein cholesterol (HDL-c), low-density lipoprotein cholesterol (LDL-C) levels, glucose (GLU), and serum UA levels. Smokers were defined as cigarette consumers who had smoked at least 20 cigarettes per week or at least 3 months per year. Drinking alcohol at least 2 times per week or at least 6 months per year was considered as alcohol consumption.

### Definition

Hypertension was defined as SBP≥140 mmHg and/or DBP≥90 mmHg, or use of antihypertensive drugs, and pre-hypertension was considered SBP 120–139 mmHg and/or DBP 80–89 mmHg.^17^ Hyperuricemia was defined as serum UA levels >4.75 mg/dL in males and >4.04 mg/dL in females.^18^ Serum UA levels were categorized by quartiles as ≤2.65, 2.66–3.24, 3.25–3.98, and ≥3.99 mg/dL.

### Data analyses

Data normality was determined using the Kolmogorov-Smirnov test. Quantitative data are summarized as mean and standard deviation (mean±SD) with normal distribution; qualitative data as proportions. Gender differences in general characteristics were analyzed using Student’s unpaired t-test for continuous variables and the Chi-square (χ2) test for categorical variables. The differences for variables among the groups were determined by one-way analysis of variance (ANOVA) or χ2 test, and Bonferroni corrections were used for multiple comparisons. Additionally, multiple unconditional logistic regression analysis was applied to estimate the relationship between UA and hypertension. Pearson’s correlation coefficient test was performed to assess the interrelationships between baseline variables and serum UA levels. Epidata 3.1 (The Epidata Association, Odense, Denmark) was used to establish databases. All statistical analyses were performed with SPSS version 18.0 (SPSS, Chicago, IL). A 2-tailed p<0.05 was defined as statistically significant.

## Results

### Participant characteristics

This study included 1,138 individuals (223 controls, 316 pre-hypertensives, and 599 hypertensive subjects) aged 35 to 75. The demographic and clinical characteristics of the participants are presented in Table 1 . The characteristics of LDL-C, creatinine, smoking, and drinking were not significantly different between the groups, whereas age, body mass index (BMI), glucose (GLU), total cholesterol (TC), triglycerides (TG), high-density lipoprotein cholesterol (HDL-C), and UA did exhibit significantly differences. Serum UA levels (mg/dL) were significantly higher in the prehypertension (3.5±1.1) and hypertension (3.4±1.1) group compared to the control group (3.2±1.0) in the entire population (p<0.05 for all). Moreover, the prevalence of hyperuricemia was 10.3%, 17.1% and 17.0% in normotensives, pre-hypertensives and hypertensives, respectively.

**Table 1:** – Demographic characteristics of normotension, pre-hypertension and hypertension

Characteristic	Normotension (n =223)	Pre-hypertension (n=316)	Hypertension (n=599)	p
Age (year)	56.1±11.3	58.2±11.2	61.2±9.8^#*^	0.000
BMI(kg/m^2^)	22.3±2.8	23.3±2.9^#^	24.1±2.9^#*^	0.000
SBP (mmHg)	108.3±7.8	126.2±7.4^#^	148.2±19.2^#*^	0.000
DBP (mmHg)	69.7±5.9	79.5±6.5^#^	89.1±13.0^#*^	0.000
GLU (mmol/L)	5.6±1.6	5.6±1.6	6.0±2.2^#*^	0.002
TC (mmol/L)	4.4±0.9	4.5±0.9	4.7±1.1^#^	0.003
TG (mmol/L)	1.2±0.8	1.3±0.8	1.7±1.2^#*^	0.000
HDL-C (mmol/L)	1.4±0.4	1.3±0.4	1.2±0.4^#*^	0.000
LDL-C (mmol/L)	2.6±0.8	2.7±0.8	2.6±0.8	0.235
Creatinine (umol/L)	82.5±61.8	80.4±13.5	86.1±39.2	0.110
UA (mg/dL)	3.2±1.0	3.5±1.1^#^	3.4±1.1^#^	0.013
Current smoker (n=362)	65 (29.1%)	95 (30.1)	202 (33.7%)	0.336
Current drinker (n=415)	68 (30.5%)	118 (37.3%)	229 (38.2%)	0.114
Prevalence of hyperuricemia (n=179)	23(10.3%)	54(17.1%)	102(17.0%)	0.046

SBP: Systolic blood pressure; DBP: Diastolic blood pressure; GLU: Glucose; TC: Total cholesterol; TG: Triglycerides; HDL-C: High-density lipoprotein cholesterol; LDL-C: Low-density lipoprotein cholesterol; UA: Uric acid. p: All participants from the normotension, pre-hypertension and hypertension groups had the variables analyzed by one-way ANOVA or Chi- square test.^#^: p<0.05 vs. Normotension.^*^: p<0.05 vs. Pre-hypertension.

By gender subgroup, of the 1,138 subjects, 568 were males, and 570 were females. The mean level of serum UA was 3.67 mg/dL in males and 3.11 mg/dL in females (p<0.05). Serum UA levels showed no difference between the groups in males. Further quantitative trait analysis of the serum UA (mg/dL) indicated that those for serum UA (2.92±0.81, 3.06±0.85, 3.22±0.98) increased linearly in normotension, pre-hypertension and hypertension in females, with a p value of 0.008 ( Figure 1 ).

**Figure 1:** – Comparison of serum UA levels in normotension, pre-hypertension and hypertension in females.

### Levels of demographic and clinical variables in the serum UA quartiles

Baseline information of the subjects in each serum UA quartile is presented in Table 2 . Mean BMI, DBP, TG, LDL-C, and creatinine were found to be increased with high levels of serum UA in the quartiles (p< 0.01 for trend).

**Table 2:** – Baseline characteristics of the study participants according to serum UA quartiles

Characteristic	Serum UA (mg/dL)				p-values for trend

	Q1(≤2.65)	Q2 (2.66-3.24)	Q3(3.25-3.98)	Q4(≥3.99)	
Age (year)	60.2±10.5	59.2±10.5	58.7±10.7	59.4±11.1	0.305
BMI (kg/m^2^)	23.1±2.8	23.1±2.8	23.5±2.9	24.3±3.2	0.000
SBP (mmHg)	133.2±23.0	136.9±21.4	132.7±22.4	134.4±20.0	0.914
DBP (mmHg)	80.5±13.2	83.4±12.3	83.0±12.7	83.7±12.8	0.005
GLU (mmol/L)	6.0±2.4	5.8±2.2	5.6±1.5	5.8±1.5	0.120
TC (mmol/L)	4.5±1.0	4.5±1.0	4.5±0.9	4.7±1.00	0.098
TG (mmol/L)	1.2±0.7	1.3±0.9	1.5±1.1	1.8±1.3	0.000
HDL-C (mmol/L)	1.3±0.4	1.3±0.5	1.2±0.4	1.2±0.4	0.266
LDL-C (mmol/L)	2.5±0.8	2.6±0.8	2.7±0.9	2.9±0.8	0.000
Creatinine (umol/L)	73.7±14.3	81.9±53.5	83.1±14.1	96.6±54.4	0.000

*SBP: Systolic blood pressure; DBP: Diastolic blood pressure; GLU: Glucose; TC: Total cholesterol; TG: Triglycerides; HDL-C: High-density lipoprotein cholesterol; LDL-C: Low-density lipoprotein cholesterol; UA: Uric acid. p: All participants from the normotension, pre-hypertension and hypertension groups had the variables analyzed by one-way ANOVA for linear trend.*

### Correlation of serum UA levels and clinical characteristics by gender

Serum UA levels were positively correlated with BMI, diastolic blood pressure (DBP), TC, TG, LDL-C, and creatinine in both genders. Serum UA levels were negatively correlated with age, and were positively correlated with BMI, TC, TG, LDL-C, and creatinine in males. In females, serum UA levels were positively associated with BMI, DBP, TG, LDL-C, and creatinine ( Table 3 ).

**Table 3:** – Correlation of serum UA levels and clinical characteristics of the study participants by gender

Characteristics	Male		Female		Total	

	r	p	r	p	r	p
Age (year)	-0.091	0.030	-0.042	0.314	-0.012	0.679
BMI (kg/m^2^)	0.167	<0.001	0.138	0.001	0.177	<0.001
SBP (mmHg)	-0.063	0.133	0.0.63	0.133	0.007	0.821
DBP (mmHg)	0.055	0.187	0.116	0.006	0.099	0.001
GLU (mmol/L)	-0.069	0.101	0.022	0.593	-0.017	0.570
TC (mmol/L)	0.152	<0.001	0.065	0.123	0.080	0.007
TG (mmol/L)	0.230	<0.001	0.205	<0.001	0.22	<0.001
HDL-C (mmol/L)	0.011	0.786	0.001	0.998	-0.041	0.163
LDL-C (mmol/L)	0.250	<0.001	0.148	<0.001	0.187	<0.001
Creatinine	0.143	0.001	0.443	<0.001	0.230	<0.001

*SBP: Systolic blood pressure; DBP: Diastolic blood pressure; GLU: Glucose; TC: Total cholesterol; TG: Triglycerides; HDL-C; High-density lipoprotein cholesterol; LDL-C: Low-density lipoprotein cholesterol; UA: Uric acid.*

### Association between serum UA quartiles and pre-hypertension and hypertension

In logistic regression analysis, Table 4 presents the odd ratios of pre-hypertension and hypertension by increasing serum UA quartiles. After adjusting for age and sex in pre-hypertension, the odd ratios (ORs) (95% CI) were 1.686 (1.024–2.775), and 2.064 (1.220–3.492), respectively in Q2 and Q4 compared to Q1. After additionally adjusting BMI, GLU, TC, TG, HDL-C, the association was still statistically significant. The second quartile of serum UA levels was significantly associated with hypertension, with an OR (95% CI) of 2.061 (1.313–3.235), and 2.036 (1.256–3.298), for models 1 and 2, respectively.

**Table 4:** – Association between serum UA quartiles and pre-hypertension and hypertension

Serum UA (mg/dL)	Pre-hypertension		Hypertension	

	Age, sex-adjusted	Multivariate	Age, sex-adjusted	Multivariate
Q1 (≤2,65)	1	1	1	1
Q2 (2.66–3.24)	1.686 (1.024–2.775)*	1.718 (1.028–2.872)*	2.061 (1.313–3.235)*	2.036 (1.256–3.298)*
Q3 (3.25–3.98)	1.091 (0.683–1.742)	1.018 (0.627–1.654)	1.105 (0.723–1.689)	0.912 (0.576–1.444)
Q4 (≥3.99)	2.064 (1.220–3.492)*	1.738 (1.003–3.010)*	2.236 (1.387–3.606)	1.613 (0.967–2.690)

Multivariate was adjusted for age, gender, BMI, GLU, TC, TG, HDL-C; * Compared to Q1, p<0.05.

## Discussion

Abnormal UA levels have been involved in vascular remodeling and endothelial dysfunction, which may be the cause of cardiovascular disorders.^19 , 20^ UA can be regarded as an important antioxidant, which doe not only stabilize endothelial nitric oxide synthase (eNOS) activity but also increases fat storage and triglycerides.^21^ Epidemiological studies have demonstrated a strong association between UA and coronary artery disease, atherosclerosis and hypertension.^22^ In our study, we report that higher serum UA levels were found to be positively associated with pre-hypertension and hypertension in middle-aged and old-age population, and high serum UA levels causes a corresponding increase in DBP. The overall risk for pre-hypertension has increased by 73.8% for the highest vs. lowest quartile of serum UA levels, even after adjusting for potential confounding variables. Furthermore, we found that the association was more robust in the female participants.

Previous studies have examined the association between serum UA levels and hypertension, and the results were in agreement with our findings. Sundstrom et al.^23^ revealed that increased serum UA levels were an independent predictor of hypertension development after a short-term follow-up. High LDL-c and serum UA levels are risk factors for endothelial dysfunction and vascular ageing. The contemporary presence of suboptimal LDL-c and serum UA values is associated with an increased risk of hypertension in an overall healthy population sample.^24^ A 5-year retrospective cohort study found that increased UA is a strong risk marker for hypertension developed from pre-hypertension in Japanese adults.^25^ Moreover, pilot clinical studies suggest lowering serum UA levels has been reported to lower blood pressure in pre-hypertensive adolescents.^26^ Currently, pre-hypertension is common in China. Approximately 20–50% of adults were affected by pre-hypertension worldwide, and this increases the risk of incident hypertension.^27^ The prevalence of pre-hypertension is rapidly increasing in China, but its causes and associated factors have not been well studied.

We observed that serum UA levels were increased linearly in normotension, pre-hypertension and hypertension in females, and this association between serum UA and blood pressure was stronger among females than in males. Besides, serum UA levels were positively associated with DBP, particularly in females. Some previous studies have demonstrated that the association between serum UA levels and hypertension was more pronounced in women. Peng et al.^28^ also found that hyperuricemia was associated with pre-hypertension among 1,773 Chinese women aged ≥30. Similar results were presented in a follow-up study, in which Strasak et al.^29^ demonstrated that serum UA is an independent predictor of all major forms of cardiovascular death in elderly women. The changing levels of serum UA in women at menopause suggests an interaction with sex hormones.^30^ Research has reported that the gender difference of blood pressure began to appear in adolescence, and pubertal growth spurt occurs earlier for girls than for boys.^31^ SBP increased significantly more in boys than in girls, while DBP increased more in girls than in boys.^32^ Other complex physiological and hormonal changes may contribute to hypertension.

Several limitations must be considered. First, the cross-sectional design used to evaluate the relationship between serum UA and pre-hypertension and hypertension limits our ability to establish a causal relationship. This problem may be solved by longitudinal studies in the future. Secondly, the interaction mechanism between hypertension and increased uric acid has not been explored. Further studies are still needed to examine the potential gender difference of the association between serum UA levels and hypertension in different populations.

## Conclusions

Our findings suggest that serum UA is significantly associated with pre-hypertension and hypertension, and the association was more robust in the female participants. Therefore, proper early management of UA levels in adults may be important to prevent the development of hypertension.
